# Quantum Dot Sensitized Solar Cell: Photoanodes, Counter Electrodes, and Electrolytes

**DOI:** 10.3390/molecules26092638

**Published:** 2021-04-30

**Authors:** Nguyen Thi Kim Chung, Phat Tan Nguyen, Ha Thanh Tung, Dang Huu Phuc

**Affiliations:** 1 Thu Dau Mot University, Number 6, Tran Van on Street, Phu Hoa Ward, Thu Dau Mot 55000, Vietnam; chungntk@tdmu.edu.vn; 2 Department of Physics, Ho Chi Minh City University of Education, Ho Chi Minh City 70250, Vietnam; phatnt@hcmup.edu.vn; 3 Faculty of Physics, Dong Thap University, Cao Lanh City 870000, Vietnam; 4 Laboratory of Applied Physics, Advanced Institute of Materials Science, Ton Duc Thang University, Ho Chi Minh City 70880, Vietnam; 5 Faculty of Applied Sciences, Ton Duc Thang University, Ho Chi Minh City 70880, Vietnam

**Keywords:** optical, electrical, photovoltaic, photoanodes, counter electrodes, electrolytes

## Abstract

In this study, we provide the reader with an overview of quantum dot application in solar cells to replace dye molecules, where the quantum dots play a key role in photon absorption and excited charge generation in the device. The brief shows the types of quantum dot sensitized solar cells and presents the obtained results of them for each type of cell, and provides the advantages and disadvantages. Lastly, methods are proposed to improve the efficiency performance in the next researching.

## 1. Introduction

Solar cells have grown very rapidly over the past few decades, which are divided into three generations: the first generation is a monocrystalline and polycrystalline Si solar cell with an efficiency of 26.7% [1] and 21.9% [2], respectively. The 2nd generation solar cells are a thin film such as CdTe [3], amorphous Si [4], the cost is lower than the 1st generation, and the efficiency is 21.7% [5]. The 3rd generation solar cells include dye sensitized solar cells (DSSCs), quantum dot sensitized solar cells (QDSSCs), perovskite cells with much lower cost than the 1st and the 2nd generation, and photoelectric conversion efficiency of over 40% according to the theoretical calculation. The highest yield obtained for DSSCs 11.9% lower than that of perovskite solar cells (19.7%) [6]. This shows the huge potential of the perovskite solar cell, it reaches an efficiency of 25.2% by 2020 [7], and it is predicted to reach 28% in the future. In addition to perovskite cells, QDSSCs are predicted to reach more than 40% efficiency according to theoretical calculations, this is also very potentially a 3rd generation solar cell.

One of the main reasons for the growing interest in quantum dots is their use in cheap solar cells, which have the possibility to increase the thermodynamic conversion efficiency above the Shockley–Queisser limit [8]. The thermodynamic limit of the light to electric power conversion efficiency, also known as Shockley–Queisser limit, originates from the fact that photons with energies below the band gap energy are not absorbed, while photons with energies above the band gap energy release the additional energy (E_photon_ − E_gap_) mostly as heat. Third generation solar cells aim for conversion efficiencies beyond the Shockley–Queisser limit through advanced photovoltaic concepts such as multijunction cells, optical up and down converters, and multiple carrier generation by impact ionization. Their development has been based on different p–n junctions and the use of quantum dots (QDs) to replace dyes. Performance above 40% has been obtained [9].

Now the focus is on the next generation solar cells with high efficiency at an economically viable cost [10,11]. QDs are drawing great attention as a material for the next generation solar cells due to high absorption coefficient, tunable band gap, and multiple exciton generation (MEG) effect [12,13]. Therefore, QDs have been used in dye-sensitized solar cells (DSSCs) as the photosensitizer instead of organic dyes to form quantum dot sensitized solar cells (QDSSCs) [14,15,16]. The typical structure of the QDSSCs is similar to that of the DSSCs, which consists of mesoporous photo anode (TiO_2_ film), sensitizer (QDs), electrolyte (polysulfide), and the counter electrode [17,18,19,20]. During operation, photons are captured by QDs, yielding electron–hole pairs that are rapidly separated into electrons and holes at the interface between the nanocrystalline TiO_2_ and the QDs. The electrons inject into the TiO_2_ film and the holes are released by redox couples in the liquid polysulfide electrolyte [17,20,21].

Improving the power conversion efficiency (η) of QDSSCs has always been an overarching concern for all scientists. One of the approaches has been focused on constructing and fabricating nanostructural oxides, such as TiO_2_ [22], ZnO [23,24], and SnO_2_ [25] to harvest more amounts of QDs. On the other hand, many efforts have been concentrated on designing and synthesizing QDs to get high photoelectric performance [26,27,28].

In recent years, researchers have discovered the QDs, which can create the high performance of solar cells [29]. QDs can be changed in particle size, leading to a change in the absorption spectrum [30]. Controlling QDs size, we can change their absorption spectrum. Furthermore, in association with biological molecules, QDs can transfer charge faster while reducing losses and helping the passivated surface (reduced defect states) of them. In 1990, Vogel and his colleagues have used CdS QDs with the Pt cathode [31]. However, this is a new direction in QDSSCs research. Since then, there have been a large number of studies such as different QDs replacement, TiO_2_ semiconductor materials, electrolyte, and counter electrodes to enhance the photovoltaic performance [32,33,34]. Lee and his colleagues studied CdSe and CdTe QDs using the Pt counter electrode with an efficiency of under 1% [35]. One year later (2008), they went on investigating CdS and CdSe QDs and improved the performance efficiency to 1.2% with the use of polysulfide electrolyte [32]. Meanwhile, Lopez-Luke et al., Mora–Sero et al., Shen et al., and Tachibana et al. [36,37,38,39] synthesized CdS and CdSe QDs with the Pt counter electrode, but in different electrolyte systems (Na_2_S, NaOH + Na_2_S+S) and obtained a better performance efficiency of 2.2%. From 2009 to 2012, various QDSSCs were studied. Cheng et al. [40] examined CdS and CdSe cosensitized TiO_2_ nanowires and nanorods by using the Na_2_S+Na_2_SO_3_ electrolyte and obtained a high efficiency of 2.41%.

Recently, a few research showed that some doping ions in the sulfide QDs, such as Hg^2+^ into PbS [41], and Mn^2+^ into CdS [42], could increase the current density and efficiency of the solar cells. Compared with CdS and PbS QDs, CdSe are more attractive owing to its high potential for light harvesting in the visible light region [43,44]. The efficiency of CdSe QDs sensitized solar cells is much higher than that of the sulfide QDs sensitized solar cells [45,46,47,48,49]. Therefore, doping metal ions into CdSe QDs is considered a useful way for designing high efficiency solar cells [50,51].

In this study, a review on QDSSCs based on photoanodes with single quantum dot, with binding agents, with passive surfactant, with multilayer QDs, and with doped QDs, different counter electrodes, and different electrolytes are briefly provided.

## 2. Quantum Dot Sensitized Solar Cells (QDSSCs) Based on Single Quantum Dots (QDs) Photoanode

### 2.1. A Review on QDSSCs Based on Single QDs

**Figure 1:**
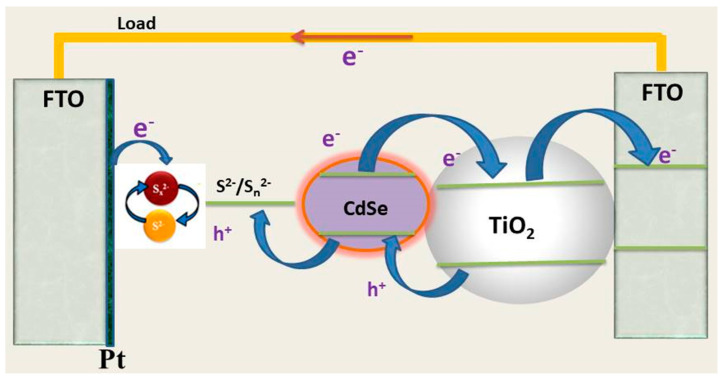
Schematic of a QDSSC’s structure with a single QDs CdSe photoanode.

Single QDs are individual QDs synthesized by several methods such as colloidal QD, chemical bath deposition (CBD), successive ionic layer absorption and reaction (SILAR), etc. Those QDs are assembled on the surface of metal oxides, which have large electronic bandgaps such as TiO_2_, ZnO, SnO_2_, etc. Both QDs and metal oxide layers are put on top of fluorine doped tin oxide (FTO) substrates to form a complete photoanode, which is illustrated in Figure 1. Thus far, there are many QDs that have been attractive globally, for example, PbS [52,53], CuInS_2_ [54], AgInSe_2_ [55], PbSeS [56], Ag_2_Se [57], CdS [58], CdSe [59], CdTe [60], etc. Among them, CdS, CdSe, and CdTe QDs are prominent candidates because of their high stability in fabricated QDSSCs [61] and achieving the highest photoelectric conversion efficiency (PCE) as illustrated in Table 1.

When a photon is absorbed, an excited electron is generated in the conduction band of a single QDs and transferred to the metal oxide layer to form a close electronic circuit. Several PCE of QDSSCs based on single QDs are shown in Table 1, whose efficiencies are relatively small, specifically, 1.31% and 1.03% [62], the highest PCE for CdS and CdSe QDs, respectively. These results are due to the absorption spectra of CdS and CdSe QDs being limited to 450 and 550 nm wavelength, respectively. This leads to the strong absorption of QDs with photons whose wavelengths are shorter than 550 nm. Otherwise, QDs are transparent to photons having wavelengths longer than 550 nm. Due to the restriction of QDs’ absorption spectra, the number of electrons produced after photo excitation is limited and greatly lost due to recombination centers (the material is imperfectly fabricated) resulting in low current density and clearly small PCE.

### 2.2. The Causes of Low QDSSCs’ PCE and Solutions

Based on the references and obtained results, there are some main reasons for the reduction of QDSSCs’ PCE, which are:

a. Low fill factor

The fill factor (FF) is defined from the current density–voltage (J–V) characteristic measurement. FF depends on the value of open circuit potential, resistance of series-connected components, and recombination processes in the QDSSCs, which relate to the fabricated materials. Low FF may be caused by the small open circuit potential, which strongly depends on the photoanode, and excessive recombination in QDSSCs. Moreover, FF is also affected by series resistors and parallel resistors of QDSSCs. To achieve higher PCE and reduce recombination in QDSSCs, it is required to have smaller series resistance R_S_ and larger parallel resistance R_SH_. From Table 1, R_S_ values of QDSSCs based on CdSe QDs are relatively large, in the range of 27.4–732 Ω. This obstructs the electrons moving through the contact layers. Similarly, relatively small R_SH_ values, which can be seen in Table 2, tend to reduce the performance of QDSSCs.

b. Impaired electrolyte

To investigate the factors that can cause a reduction in QDSSCs’ PCE, the reduced absorption ability of the electrolyte after photoanode immersion is studied. According to Kamat et al., the electrolyte is in direct contact with the TiO_2_/CdSe membrane, so, in operation, CdSe QDs react with the electrolyte and generate byproducts, which impair both the electrolyte and CdSe QDs. Those reactions are described by the following equations:

- A dynamic balance is existed in the S^2−^/S_n_^2−^ aqueous electrolyte:

S^2−^ + H_2_O ↔ HS^−^ + OH^−^ (1)

- Electron–hole pairs are generated after CdSe QDS are photoexcited by possibly the following equations:

CdSe + hν → CdSe (e + h) → CdSe + hν’(2)

CdSe (e + h) + TiO_2_ → CdSe (h) + TiO_2_ (e) (3)

- Reaction at the CdSe/electrolyte interface:

CdSe (h) + S^2−^ → CdSe + S^−*^ (4)

- The S^2−^/Sn^2−^ strongly obstructs the hole movement from CdSe QDs into the electrolyte [27] as described in Equation (4) and S^−*^ is in an excited ion.

**Figure 2:**
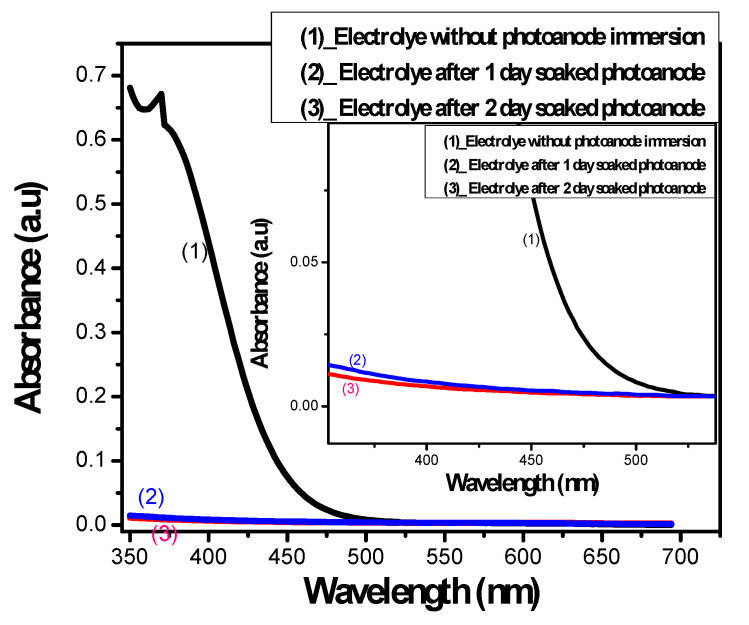
The absorption spectrum of polysulfide electrolyte before and at specific times after photoanode immersion.

As can be seen in Figure 2, the absorption of the electrolyte was dramatically decreased after 2 days of immersion. This proves the large influence of the electronic exchange reactions between CdSe QDs and the electrolyte, as generated byproducts from Equations (1)–(4) impaired the electrolyte absorbance and reduced the QDSSCs’ PCE.

c. Strong recombination processes

**Figure 3:**
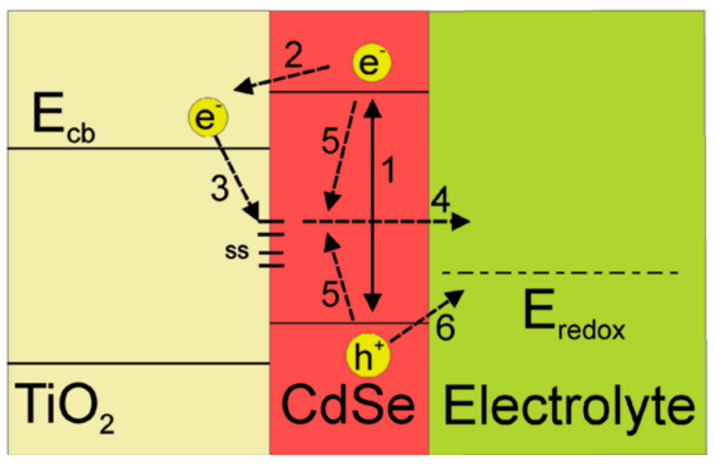
Schematic of the energy levels of different material layers and main processes in an operating QDSSC: (1) exciton generation in CdSe QDs, (2) electrons transferring from CdSe QDs into TiO_2_ layer, (3) electron trapping causing by QDs’ surface trap states, (4) electrons diffusion from CdSe QDs into electrolyte, (5) relaxation in CdSe QDs, and (6) recombination of electrons in electrolyte and holes in CdSe QDs by reduction reaction at the QDs/electrolyte interface. It was obtained from Mora-Sero and co-works, 2009 [79].

A typical structure of QDSSCs includes the photoanode, counter electrode, and polysulfide electrolyte. Under the light condition, the operated processes happening inside QDSSCs are shown in Figure 3. These processes are indicated by arrows. When the photoanode surface is illuminated, exciton generation and electron–hole recombination are exhibiting simultaneously inside CdSe QDs (1). Free electrons in the conduction band of CdSe QDs are easily transferred to the TiO_2_ conduction band (2). Those electrons, however, may be trapped in surface states due to CdSe QDs imperfection (3) and diffused into the electrolyte afterward (4), or recombined with holes inside CdSe QDs. The recombination of free electrons in the conduction band (CB) with CdSe QDs surface state and the recombination of electrons in the electrolyte and holes in CdSe QDs valence band (VB) are indicated as (5) and (6) processes, respectively. The (1), (2), and (6) processes are useful in QDSSCs operation. In contrast, the remaining processes cause a reduction in QDSSCs’ performance.

d. Shortage of binding agents between QDs and TiO_2_ membrane

Among QDSSCs, which are prepared based on TiO_2_, the membrane is soaked directly into TOP organic solvents, where CdSe QDs are dissolved. The solvents, however, create unsustainable chemical bonding with TiO_2_ molecules. Consequently, it reduces the carrier transport efficiency in QDSSCs.

## 3. QDSSCs Based on Photoanode Binding Agents

**Figure 4:**
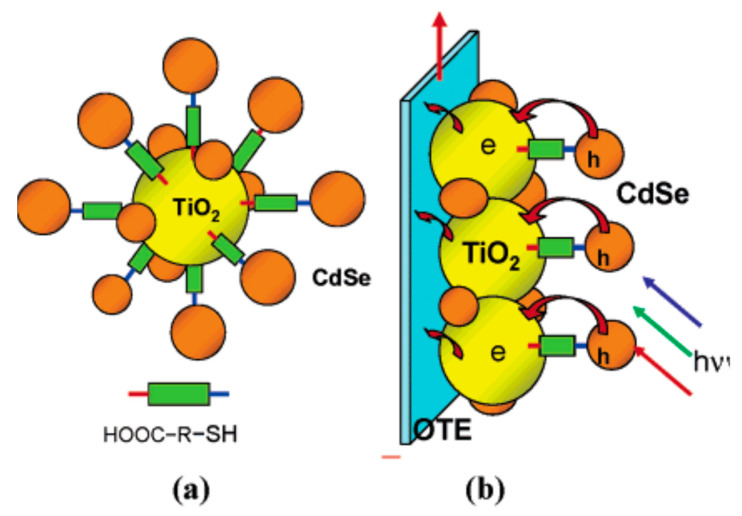
(**a**) The absorption of single CdSe QDs onto the metal oxide membrane supported by binding agents and (**b**) photoanode is formed by coating system (**a**) with the FTO glass substrate [84].

The single QDs presented in the last section are directly absorbed onto the metal oxide membrane. It is difficult to achieve perfect absorption at the QDs/TiO_2_ interface, hence electronic trap states have consequently arisen. To reduce the recombination at QDs/metal oxide interface, binding agents, such as mercaptopropionic acid (MPA), trioctylphosphine, or trioctylphosphine oxide [82,83], thiolacetid acid (TAA), or mercaptohexadecanoic acid [84], are frequently used. With the formation of COOH-R-SH, the COOH- (carboxyl) group of binding agents can establish chemical bonding with metal oxides, while -SH (thiol) group is linked to single QDs, as illustrated in Figure 4. The presence of binding agents supports the electron transport from the CB of QDs into the metal oxide layer, consequently, the current density enhancement in QDSSCs has been observed.

## 4. QDSSCs Based on a Photoanode with a Passive Surfactant

The actual PCE of the majority of QDSSCs, which have a single QDs photoanode, is less than from the theoretical calculation due to the strong recombination inside QDs and at QDs/TiO_2_ interface or direct contact of QDs with the different electrolyte systems. To limit the number of surface trap states, materials with large band gaps, such as ZnS, SiO_2_, MgO, or Al_2_O_3_ (shown in Table 3), have been frequently used as a passivation layer [85,86,87,88,89,90]. The passivation layer is covered on the surface of QDs to create a boundary that prevents the direct contact of QDs with the electrolyte and hence the dark current, i.e., stimulated electron transport from QDs to the electrolyte. One can imagine that the excited electrons are blocked by the presence of this passivation layer and only able to move from QDs to TiO_2_ and then to the outer circuit.

**Figure 5:**
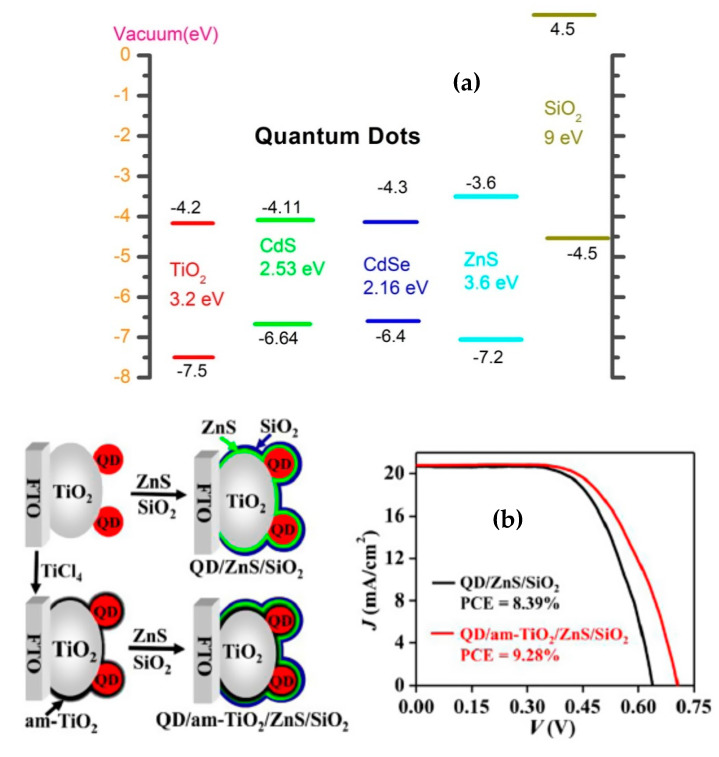
(**a**) Schematic diagram of the energy level of an QDSSC’s photoanode [101] and (**b**) the enhancement in QDSSCs performance by using a passive surfactant from Ren and co-work, 2015 [105].

Currently, ZnS has been widely used as the most effective passive surfactant for QDSSCs since this semiconductor has a large band gap of approximately 3.6 eV and CB level (−3.6 eV) of nanocrystal ZnS is higher than that of CdSe (−4.3 eV) and CdS (−4.11 eV), which is studied by Tung et al. and illustrated in Figure 5a. The efficiency was significantly increased by 150% with the presence of a passivation layer. In addition, Tung and colleagues reported an efficiency enhancement from 1.64% to 3.77% on QDSSCs based CdS/CdSe:Mn photoanode and from 1.64% to 4.22% on CdS/CdSe:Cu-based photoanode with the presence of ZnS passivation layer [99,100,101,102]. As reported by Hachiya et al. [95], a significant improvement of excited electron transport from QDs into TiO_2_ layer and a dramatic reduction of surface trap states at the QDs/TiO_2_ interface by covering ZnS nanocrystals on PbS QDs were observed and proved by transient grating (TG) spectra. Apart from ZnS, other materials, such as ZnSe [103], Al_2_O_3_ [104], or SiO_2_ [101], have been used as a passivation layer for QDSSCs. According to Tung et al., the QDSSCs efficiency was sharply increased by 375% when a SiO_2_ passivation layer was absorbed onto the surface of CdS/CdSe QDs. Since SiO_2_ conduction level in a vacuum (4.5 eV) was much higher than those of CdSe (−4.3 eV) and CdS (−4.11 eV), the dark current was mostly suppressed in QDSSCs leading to the PCE enhancement. Furthermore, the combination of ZnS and SiO_2_ as a passivation layer has shown promising efficiency in recent studies. The passivation layer of ZnS/SiO_2_ covered on the surface of CdSe_x_Te_1−x_ QDs, in particular, leads to the significantly increased efficiency from 6.37% to 8.55% [103].

## 5. QDSSCs Based on a Photoanode with Multilayer QDs

QDSSCs based on single QDs have limited absorption spectra in the visible region. To overcome CdS, CdSe, CdTe, and PbS QDs are combined to be able to absorb photons with different wavelengths in the visible region. Recently, photoanodes with multilayer QDs, such as CdS/CdSe [105], CdS/CdTe [106], CdS/PbS [107], CdS/CdSe/PbS [108], CdSe/CdTe [109], or ZnTe/CdSe [110], have been studied. Osada et al. reported a 70% increasement in PCE of QDSSCs by covering a CdS layer prior to TiO_2_, i.e., CdS acts as a buffer layer, while only 50% enhancement with CdSe prior covering. This result has good agreement with others reported by Tung and colleagues [111]. Specifically, they observed a raise from 0.6% to 1.05% in QDSSC’ PCE when a CdS layer is sandwiched between TiO_2_ and CdSe outer layers. Several publications on the electric transport researched inside QDSSCs based on TG spectra have proved that the tandem (or parallel or cophotosensitive) structure, as can be seen in Figure 5a, leads to more photon absorption and hence more exciton generation [112,113].

**Figure 6:**
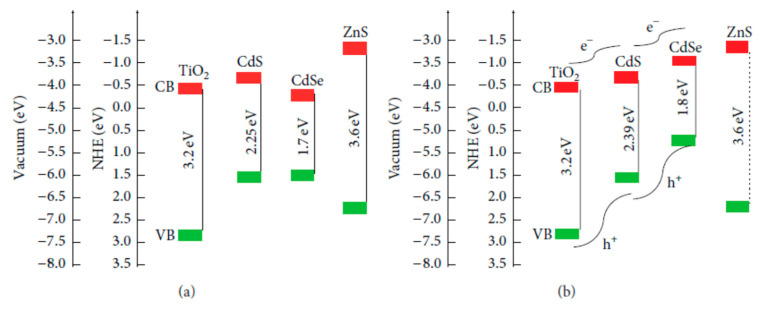
Schematic diagram of the energy level of a photoanode with (**a**) CdS CdSe in bulk scale and (**b**) CdS CdSe in the nanoscale from Grätzel, 2001 [114].

In Figure 6a, TiO_2_, CdS, and CdSe are in bulk scale, so the CB level of bulk CdSe is lower than those of bulk TiO_2_ and CdS. This structure obstructs the electron movement from the CB of CdS and CdSe QDs into the TiO_2_ layer. However, when CdS and CdSe are in the nanoscale, their band gap can be manipulated. As can be seen in Figure 6b, the band gaps of CdS and CdSe nanocrystals were 2.39 and 1.8 eV, respectively. Due to quantum confinement, the conduction energy levels of both CdS and CdSe nanocrystals were higher than that of bulk TiO_2_. This yielded a tandem structure at the photoanode energy, which is favorable for electron transport from QDs into TiO_2_. Moreover, with the tandem structure shown in Figure 6, light propagates in order through FTO, TiO_2_, CdS, CdSe, and ZnS layers. FTO glass substrate is transparent, so light energy is preserved when reaching the TiO_2_ layer. Due to the large bandgap of 3.2 eV, the TiO_2_ layer just absorbed photons with wavelengths less than 400 nm. Other longer wavelength photons were continuously propagated and then those with wavelengths less than 450 nm and 650 nm were absorbed by CdS and CdSe QDs, respectively. Therefore, the tandem structure of photoanode energy led to a broader absorption spectrum from the ultraviolet region to 650 nm in the visible region and more exciton generation, consequently, PCE of QDSSCs may be enhanced. However, the excited electrons can be trapped by a number of surface states at TiO_2_/CdS/CdSe/ZnS interface due to the imperfect synthesis process, which affects the performance of QDSSCs [100].

## 6. QDSSCs Based on a Photoanode with Doped QDs

In recent years, CdSe QDs has been attractively researched and applied in QDSSCs fabrication due to their simple synthesis, low cost, and high chemical stability. The resistance, however, of CdSe was relatively high and the CB level of this material in the bulk state was slightly slower than that of TiO_2_, which obstructed the movement of photoexcited electrons from the CB of CdSe QDs into TiO_2_. To improve the current density and hence the PCE of QDSSCs, several research have been carried out on doping metals, such as Mg [115], Mn [99], Cu [100], Ag [116], Hg [117], Co [118], or Eu [119], into CdS, CdSe or PbS QDs. 

**Figure 7:**
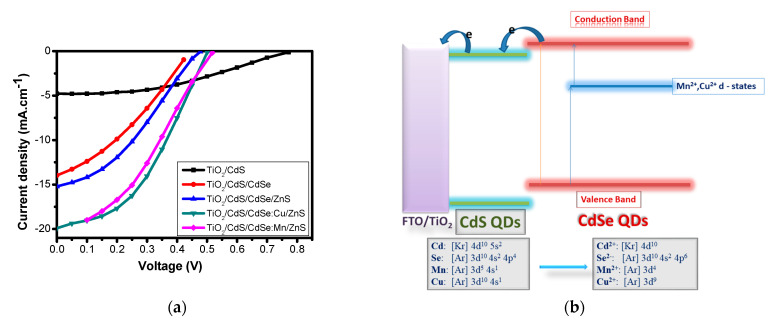
(**a**) Current density–voltage (J–V) curves of QDSSCs with different photoanodes and (**b**) schematic diagram of the energy level of a photoanode with CdSe QDs doped Mn or Cu from Phuc, D.H and co-works, 2019 [120].

As reported by Tung et al., by doping Mn and Cu into CdSe QDs, the efficiencies of QDSSCs were increased from 2.55% with pure tandem-structure photoanode to 3.77% and 4.22% with Mn- and Cu-doped photoanode, respectively. These results have good agreement with those in Reference [97]. The current density enhancement in QDSSCs with the doped photoanode was due to the presence of doping metal energy level in the bandgap of pure QDs, as can be seen in Figure 7b. Hence, one can manipulate the bandgap of QDs, for instance, CdSe:Cu^2+^ QDs, by controlling the doping concentration and layer thickness. Without doping, photons whose energy was less than the pure QDs bandgap cannot be absorbed. However, by the presence of doping energy levels inside the pure QDs bandgap, those aforementioned photons were able to be absorbed. This led to a significant improvement of the photoexcited electron density and hence the current density of QDSSCs [120,121,122]. Moreover, by doping metals into pure QDs, the resistance of different components in QDSSCs, such as TiO_2_/QDs interface and TiO_2_ diffusion layer resistance (R_ct2_) or electrolyte/counter electrode resistance (R_ct1_), were dramatically decreased, while the significant increasement of photoexcited electron lifetime in the QDs CB was observed [100].

## 7. QDSSCs Based on Different Counter Electrodes

**Figure 8:**
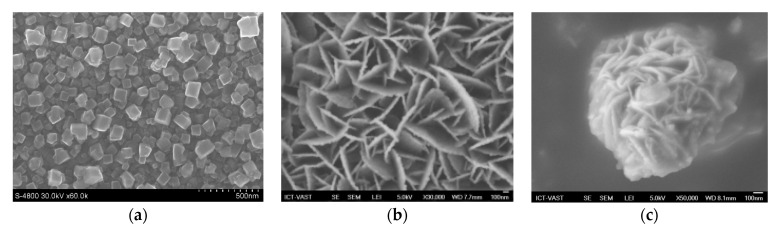
FE-SEM images of counter electrodes based on different materials: (**a**) PbS, (**b**) CuS, and (**c**) Cu_2_S from Tung HT and co-works, 2014 [129].

**Figure 9:**
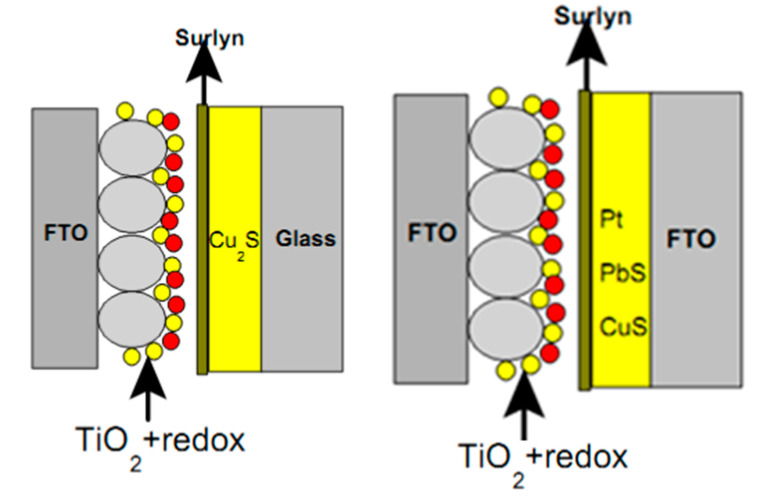
Schematic diagram of QDSSCs’ structure with different counter electrode materials: Cu_2_S (**left**) and Pt, CuS, and PbS (**right**).

Counter electrode has a large contribution on the operation of QDSSCs, so choosing the suitable material for counter electrode fabrication is strongly required. The reduction reaction of the electrolyte system is occurred at the counter electrode surface, hence the counter electrode material must have low resistance and high electrochemical catalyst activity, i.e., to reduce the redox potential of the electrolyte. Pt was previously used as the cathode material due to its compatibility with the I^3−^/I^−^ electrolyte as a Figure 9. However, the resistance of the counter electrode/electrolyte interface is relatively high, which reduces the electron transport efficiency through the cathode surface [123]. For QDSSCs, QDs are easily corroded in the I^3−^/I^−^ electrolyte and hence limit the light absorption ability of those QDs. Several cathode materials, such as nanocarbon tubes, CuS, nanocarbon tube–Cu_2_S, NiS, or Au (Figure 8 and Figure 9) [124,125,126], have been studied in QDSSCs with a polysulfide electrolyte. Among them, nanocarbon tube, Au and NiS are suitable for the polysulfide electrolyte but have high resistance at the counter electrode/electrolyte interface, hence limiting the electron movement from counter electrode to electrolyte and reduce the efficiency of QDSSCs. CoS and NiS materials have been studied for better results, but they leave impurities in the electrolyte and counter electrode, which affect QDSSCS in the long-term operation [127,128]. 

**Figure 10:**
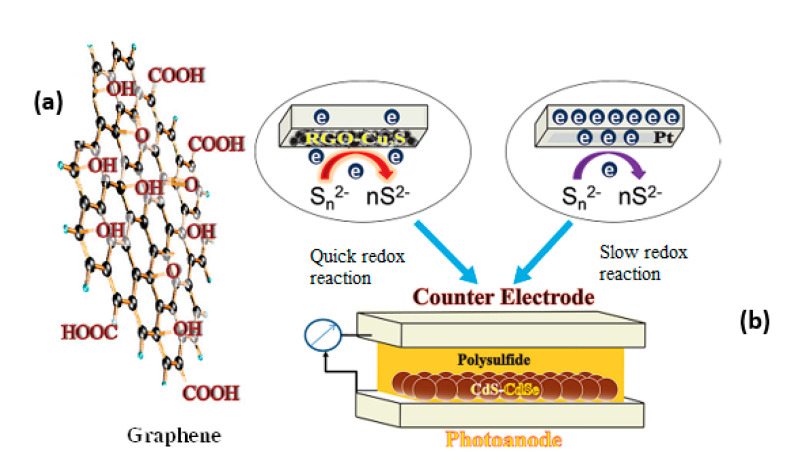
(**a**) Graphene with functional groups and (**b**) structure of a QDSSC from Tachan and co-works, 2011 [133].

Graphene is a two-dimensional single layer of carbon atoms, which has a large surface area and high electronic mobility of 1.5 × 10^4^ cm^2^ V^−1^ s^−1^ so having prominent electrical, optical, thermal dynamics, and mechanical properties [130,131,132]. Therefore, graphene has become promising for science and technology revolution. Moreover, graphene oxide is more applicable due to possessing -COOH and -OH function groups lying between horizontal lattices and on the corner of the horizontal plane, which can form carbonyl or carboxylic (Figure 10) to easily establish chemical bonding with inorganic materials, such as Cu_x_S, on counter electrodes of QDSSCs.

Cu_x_S and its compounds have superior absorption and decompose chemical reaction ability, which lead to efficiency enhancement, so they have been widely used as counter electrode materials in QDSSCs [134]. Lee et al. studied the composite of Cu_2_S and nanocarbon tube, but only 0.08% efficiency was achieved due to the carrier mobility of the nanocarbon tube is much lower than that of graphene [135]. 

## 8. QDSSCs Based on Different Electrolytes

As discussed in Section 6, QDSSCs derived from the previous type of solar cells, which had dye molecules as photo absorbers, so keep using the I^−^/I^3−^ electrolyte. This electrolyte, however, is the main cause of corrosion and functional degradation of QDs and hence low efficiency, for instance, 1.52% PCE of QDSSCs based on CdS/CdSe QDs [123]. Therefore, seeking for a more compatible electrolyte with QDs to improve QDSSCs performance is the main challenge. The electrolyte, according to recent reports (Table 4), can now be divided into three categories, which are liquid, pseudo-solid, and solid electrolytes. 

Among them, liquid polysulfide electrolyte has been widely used due to its compatibility with QDs and counter electrodes, which greatly improve the QDSSCS performance [144]. This electrolyte, unfortunately, causes oxidation of the QDs and makes the open circuit potential (V_OC_) and FF low [145]. To protect from corrosion, QDs are frequently covered by a layer of passivation surfactants, such as ZnS or SiO_2_, as discussed above. In addition to the polysulfide electrolyte, the pseudo-solid [146] or solid [147] electrolyte has been used in the combination with organic compounds, such as polyethylene glycol [148] and guanidine thiocyanate [149].

## 9. Opportunities and Challenges

Currently, QDs have been made with high purity, quality, less defects, manipulated bandgap energy, and good optical properties to replace photosensitive molecules. However, the PCE of QCSSCs has not exceeded 10%, which is much lower than that from theoretical calculations. There are two major issues one has to overcome:

Firstly, substantial excitation electron loss in QDSSCs. There are many types of losses in QDSSCs operation, such as the recombination process inside QDs or on their surfaces due to imperfect fabrication, electron loss when transported through the QDs/TiO_2_ interface, electron diffusion process inside the TiO_2_ membrane, and QDs corrosion by electrolyte or electron loss due to redox reaction at the electrolyte/counter electrode interface. Among them, the losses due to internal and surface defects of QDs are limited for those QDs synthesized by the colloidal process at high temperature or extended for those made by the CBD or SILAR method. Losses due to electrolyte corrosion can be improved by using passivation surfactants, such as ZnS or SiO_2_, to limit the contact between QDs and electrolytes. However, more defects are observed in the multilayer photoanodes with tandem structure.

Secondly, researching on the new kind of QDs materials.

a. QDSSCs based on QDs possessing intermediate band (IB)

**Figure 11:**
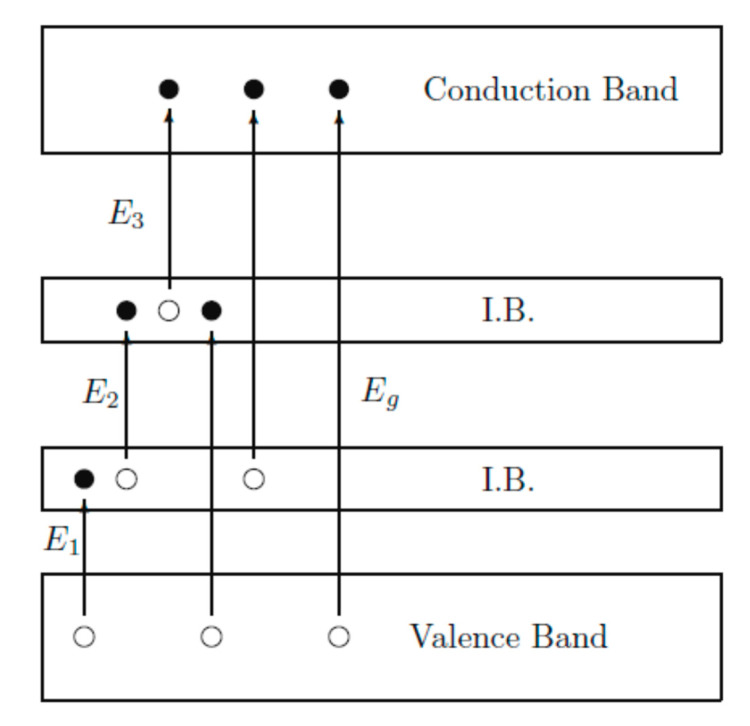
Schematic diagram of energy levels of an IB structure material from Wu and co-works, 2012 [151].

Limitations in traditional solar cells, such as a narrow absorption spectrum of photosensitive materials, leads to the degradation of absorbed photon density, current density, and open circuit potential. Those photons, which have energy larger or equal than the photosensitive material bandgap, are absorbed. For materials with IB structure, this issue is overcome since the material is a compound of two or more materials with different bandgap energies. As can be seen in Figure 11, photons with different energies E_1_, E2, and E_3_ are absorbed, corresponding to the energy gap between two VBs of the two materials, valence and CBs of the narrower bandgap material and two CBs of the two materials, respectively. These absorptions result in the enhancement of the excited electron concentration in the CB of a wider bandgap material, which is then collected and transferred to an external circuit and creates the electric current density. These IB structure materials have great potential to replace traditional photosensitive materials due to predicted PCE in QDSSCs up to 46% [150].

b. Graphene thin film with QDs for photoanode fabrication

Graphene is a two-dimensional single layer of carbon atoms, which has a large surface area and high electronic mobility of 1.5 × 10^4^ cm^2^ V^−1^ s^−1^ so having prominent electrical, optical, thermal dynamics, and mechanical properties [151,152,153]. Carbon-based graphene materials have outstanding properties, especially graphene quantum dots with extremely effective electron transport properties, and other interesting phenomena due to the quantum confinement effect [154]. Therefore, graphene has become promising for science and technology revolution. Several reports on this kind of material have been observed. Dutta et al. synthesized graphene QDs and then absorbed onto the surface of ZnO fibers by chemical deposition method, resulting in a 0.8 V open circuit potential [154]. Zhong and colleagues combined graphene QDs with CdSe QDs covering the TiO_2_ membrane for 6% conversion efficiency [155]. Graphene QDs combined with other QDs, such as CdSe, CdS, or CdTe, is an effective method to improve PCE of QDSSCs.

**Figure 12:**
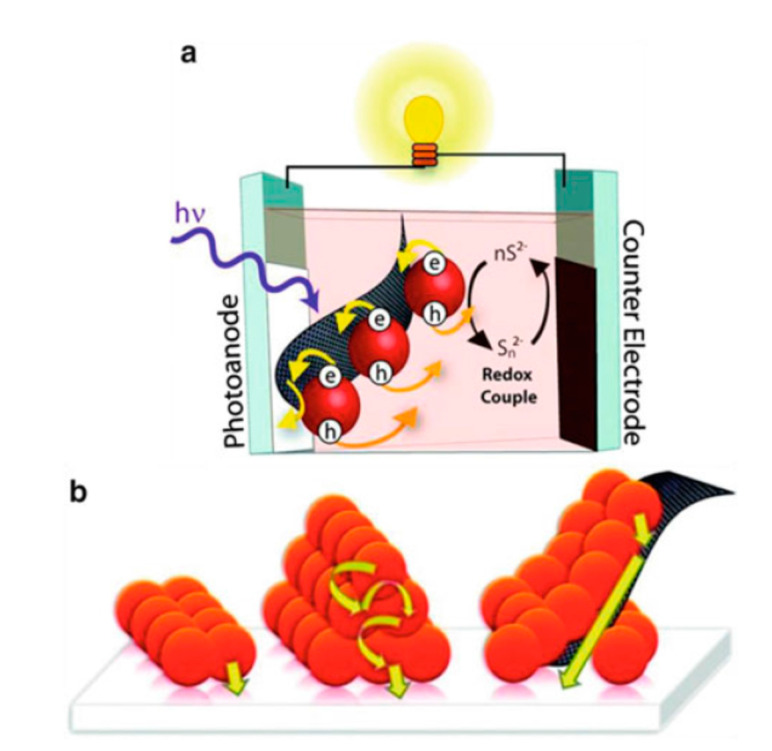
(**a**) Structure of QDSSCs based on graphene/CdSe QDs photoanode and (**b**) schematic of a photoanode that has graphene/CdSe QDs absorbing on the FTO substrate.

Figure 12 shows a schematic diagram of a QDSSCs based on a photoanode with graphene. Core–shell structure CdSe/ZnS QDs absorbed on a graphene membrane has demonstrated the faster electron transportation from QDs to the TiO_2_ layer [156]. The superiority of graphene is demonstrated in another report, which studied the transient absorption spectrum of pure and CdTe QDs absorbed graphene resulting in a significant increase of relaxation time from 50 ps in pure QDs to 200 ps in absorbed graphene and hence increases the excitation electron density in the CB of QDs and is the basis for increasing the current density in QDSSCs [157].

## Figures and Tables

**Table 1:** Review of the same field publications.

Metal Oxide Layer	QDs	Counter Electrode	Electrolyte	Synthesis Method	FF (%)	PCE (%)	Ref
TiO_2_	CdS	Pt	KCl+Na_2_S	SILAR	-	-	[61]
TiO_2_	CdSe	Pt	[Fe(CN)_6_]^3−/4−^	CBD	-	-	[62]
TiO_2_	CdSe	Pt	Na_2_S+Na_2_SO_4_	CBD	-	-	[63]
TiO_2_	CdS	Pt	Na_2_SO_3_	CBD	-	-	[64]
TiO_2_	CdSe	Pt	Polysulfide	CBD	59	1.03	[65]
TiO_2_	CdSe	Pt	Na_2_S	CBD	40	0.7	[66]
TiO_2_	CdSe	Pt	Na_2_S	CBD	27.7	0.84	[67]
TiO_2_	CdSe	Pt	Na_2_S+S+NaOH	CBD	43	0.4	[68]
TiO_2_	CdS	Pt	LiI+I_2_+DMPII+TPB	CBD	70	0.3	[69]
TiO_2_	CdSe	Pt	LiI+I_2_+HMII+TPB	CBD, Linker	56.3	1.19	[70]
TiO_2_	CdS/CdSe	Pt	Na_2_S+S	CBD	41.5	1.42	[71]
TiO_2_	CdS	Pt	KCl+Na_2_S	SILAR	-	-	[72]
TiO_2_	CdS/CdSe	Pt	Na_2_S+S+KCl	SILAR	36	1.14	[73]
TiO_2_	CdS/ZnSe	Pt	Thiourea	CBD, Linker	58	0.86	[74]
TiO_2_	CdS/CdSe	Pt	Na_2_S+S+KCl	CBD	37	1.33	[75]
TiO_2_	CdS/CdSe/ZnS	CuS, CoS	Polysulfide	SILAR	35	2.7	[76]
TiO_2_	CdS/ZnS	Pt	Sulfide	SILAR	46	1.72	[77]
TiO_2_/ZnS	CdS/CdSe	Cu_2_S	Na_2_S+S	SILAR	66	4.21	[78]
TiO_2_	CdS/CuInS_2_	Carbon	Na_2_S+S	Colloid	37	1.47	[79]
TiO_2_	CdS/JK24	Pt	Na_2_S+S	Colloid	38.2	1.18	[80]
Graphene-TiO_2_	CdS	Pt	Na_2_S+S	Colloid	41	1.31	[81]
TiO_2_	CdS/CdSe	Pt	Na_2_S+S+KCl	SILAR	36	1.14	[73]
TiO_2_	CdS/ZnSe	Pt	Thiourea	CBD, Linker	58	0.86	[74]

**Table 2:** The resistance values of QDSSCs as calculated by physical approach.

QDSSCs	R_D_ (Ω)	R_d_ (Ω)	R_S_ (Ω)	R_SH_ (Ω)	PCE η (%)
1-h soaked TiO_2_/CdSe	1230.0	498.0	732.0	239.0	0.020
10-h soaked TiO_2_/CdSe	538.2	382.0	156.2	588.1	0.046
18-h soaked TiO_2_/CdSe	157.3	83.1	74.2	2027.0	0.184
20-h soaked TiO_2_/CdSe	60.8	33.2	27.4	5396.0	0.575
24-h soaked TiO_2_/CdSe	136.5	80.0	56.5	2130.0	0.150

**Table 3:** Review on QDSSCs with passive surfactant.

QDs	Passivation Layer	PCE (%)	References
CdSe	ZnS	2.1	[91]
CdS/CdSe	ZnS	4.92	[92]
CdSe_x_Te_1-x_	ZnS/SiO_2_	8.55	[93]
PbS/CdS	ZnS	4.2	[94]
PbS/CdS	ZnS/SiO_2_	7.19	[95]
CdTe/CdSe	ZnS	6.76	[96]
ZnTe/CdSe	ZnS	6.82	[97]
CdS/CdSe	ZnS	2.07	[98]
CdS/CdSe:Mn	ZnS	3.77	[99]
CdS/CdSe:Cu	ZnS	4.22	[100]

**Table 4:** Review on QDSSCS based on different counter electrodes.

Counter Electrode	QDs	PCE (%)	References
Pt	CdS/CdSe	1.52	[123]
Au	CdS/CdSe	4.22	[136]
Cu_x_S	CdSe_x_Te_1−x_	9.28	[137]
Cu_x_S	CdSe_x_Te_1−x_	8.72	[138]
Cu_2_S	CdSe_x_Te_1−x_	6.12	[139]
CoS	CdS/CdSe	4.16	[140]
Cu_2_S	CdS/CdSe:Cu	4.22	[100]
rGO-Cu_2_S	CdS/CdSe	4.4	[141]
NiS	CdS	2.5	[142]
NiS_2_	CdS/CdSe	2.25	[143]
PbS	CdS/CdSe	3.91	[144]
